# Regulatory T Cells from Patients with Rheumatoid Arthritis Are Characterized by Reduced Expression of Ikaros Zinc Finger Transcription Factors

**DOI:** 10.3390/cells11142171

**Published:** 2022-07-11

**Authors:** Mara Dittrich-Salamon, Anja Meyer, Shuaifeng Yan, Eva Steinbach-Knödgen, Konstantin Kotschenreuther, David Stahl, Carola tho Pesch, Joanna Schiller, Franziska Byrtus, Dorothee Jochimsen, Viktoria Golumba-Nagy, David M. Kofler

**Affiliations:** 1 Laboratory of Molecular Immunology, Division of Rheumatology and Clinical Immunology, Department I of Internal Medicine, Faculty of Medicine and University Hospital Cologne, University of Cologne, Kerpenerstr. 62, 50937 Cologne, Germany; ditt.sala@gmail.com (M.D.-S.); ameyer1711@gmail.com (A.M.); shuaifeng.yan@uk-koeln.de (S.Y.); eva.steinbach-knoedgen@uk-koeln.de (E.S.-K.); kkotsche@smail.uni-koeln.de (K.K.); viktoria.golumba-nagy@uk-koeln.de (V.G.-N.); 2 Division of Rheumatology and Clinical Immunology, Department I of Internal Medicine, Faculty of Medicine and University Hospital Cologne, University of Cologne, Kerpenerstr. 62, 50937 Cologne, Germany; david.stahl1@uk-koeln.de (D.S.); carola.tho-pesch@uk-koeln.de (C.t.P.); joanna.schiller@uk-koeln.de (J.S.); franziska.byrtus@uk-koeln.de (F.B.); dorothee.jochimsen@uk-koeln.de (D.J.); 3 Center for Integrated Oncology Aachen Bonn Cologne Duesseldorf, Kerpenerstr. 62, 50937 Cologne, Germany

**Keywords:** rheumatoid arthritis, regulatory T cells, Ikaros, Helios, Aiolos, Eos, Th1 cells, Th17 cells, disease activity, DAS28-CRP

## Abstract

Regulatory T (Treg) cells play an important role in immune tolerance and contribute to the prevention of autoimmune diseases, including rheumatoid arthritis (RA). The differentiation, function and stability of Treg cells is controlled by members of the Ikaros zinc finger transcription factor family. In this study, we aimed to reveal how the expression of Ikaros transcription factors is affected by disease activity in RA. Therefore, we analyzed the ex vivo expression of Ikaros, Helios, Aiolos and Eos in Treg cells, Th17 cells and Th1 cells from RA patients by flow cytometry. We found significantly reduced expression of Helios, Aiolos and Eos in Treg cells from RA patients as compared to healthy controls. Moreover, Helios and Aiolos levels correlated with disease activity, as assessed by DAS28-CRP. In addition, Ikaros, Helios and Aiolos were significantly downregulated in Th1 cells from RA patients, while no difference between healthy individuals and RA was observed in Th17 cells. In summary, Helios and Aiolos expression in Treg cells correlates with disease activity and the expression levels of Ikaros transcription factors are diminished in Treg cells from RA patients. This observation could explain the reduced stability of Treg cells in RA.

## 1. Background

The Ikaros family of transcription factors consists of five zinc finger proteins: Ikaros (encoded by *IKZF1*), Helios (*IKZF2*), Aiolos (*IKZF3*), Eos (*IKZF4*) and Pegasus (*IKZF5*) [1,2,3]. The transcription factors Ikaros, Helios, Aiolos and Eos are implicated in the differentiation of B cells and T cells [4,5]. So far, no specific role of Pegasus has been identified in hematopoietic cells. Aiolos and Helios regulate the development and the stability of Treg cells. Deficient or altered expression of Aiolos in T or B cells is found in various diseases, including chronic lymphocytic leukemia, pneumocystis pneumonia, systemic lupus erythematosus and rheumatoid arthritis (RA) [6,7,8,9]. Aiolos promotes Th17 cell induction by directly silencing *IL2* [10]. Interestingly, deficiency of Aiolos inhibits Th17 cell development and drives the expansion of Th1 cells [10]. Furthermore, Aiolos is associated with IL-10 expression in tumor necrosis factor-α (TNF-α) inhibitor-exposed Th17 cells [11]. However, Aiolos is not sufficient for IL-10 production by CD4+ T cells, which is inhibited by degradation of Aiolos but not increased by overexpression of Aiolos [12]. In addition to its role in Th17 cell differentiation, Aiolos is required for induction of functional FoxP3+ iTreg cells [13,14]. These induced Treg cells express the interleukin-1 receptor RI (IL-1RI) and the L-phenylalanine IL4I1, but not Helios [13,15].

Ikaros acts as a negative regulator of Th1 cells by silencing T-bet and inhibiting IFN-γ production [16]. Moreover, Ikaros has been linked to Treg cell differentiation [17]. Mice with Ikaros deficiency have reduced numbers of natural Treg cells as compared to wildtype mice [17]. Remarkably, Ikaros deficiency can be induced by the drug iberdomide [18]. Helios and Eos have been reported to stabilize the suppressive phenotype of FoxP3+ Treg cells and to determine their suppressive capacity [15,19,20,21,22,23]. The role of Eos in Treg cells has been demonstrated by selective deletion in Treg cells, which leads to loss of suppressive functions and to the development of autoimmune diseases [24]. Eos^−/−^ mice develop more severe experimental autoimmune encephalomyelitis (EAE) as compared to wildtype (WT) mice, underlying its role as an immune suppressor [25]. However, a certain redundancy of Eos has been postulated because Treg cells from Eos^−/−^ mice show the same ability to suppress effector T cells as Treg cells from WT mice [25]. Interestingly, downregulation of Eos is mechanistically required in order for Treg cells to undergo reprogramming, while FoxP3 expression remains stable in reprogrammed Treg cells [21]. The suppression of Eos in Treg cells has been reported to be mediated by IL-6, a key player in the pathogenesis of RA [21]. IL-6 and soluble IL-6 receptor (IL-6R) levels are elevated in the serum of patients with RA and correlate with disease activity [26].

RA is a common systemic autoimmune disease with a complex pathogenesis. The activation status of T cells in RA is modified by various environmental and genetic factors [27,28,29,30]. Dysfunction of Tregs is one of the proposed mechanisms underlying the breakdown of self-tolerance in RA [31]. The balance between Treg cells and Th17 cells is disturbed in RA, resulting in a shift towards Th17 cells and increased Th17 cell frequencies in the peripheral blood [32,33]. Furthermore, the migratory capacity of Treg cells is reduced in RA [32,34]. Studies about the frequency of Treg cells in the peripheral blood of RA patients have reported contradictory results, showing either increased or decreased Treg cell numbers in RA [31,35]. The discrepancy between these results can be explained by different strategies used to identify human Treg cells. Most studies using the CD25^high^CD127^low^FoxP3^+^ phenotype to characterize Treg cells have reported decreased Treg cell frequencies in RA [35]. Importantly, treatment with classical or biological disease-modifying anti-rheumatic drugs (DMARD) can restore Treg cell frequencies in the peripheral blood of patients with RA [36]. 

The expression and function of Helios and Eos in Treg cells from patients with RA have been studied recently. Helios has been suggested as a marker for functional Treg cells in patients with RA [37,38]. Moreover, Helios enhances the function of induced Treg cells from RA patients in cooperation with FoxP3 [39]. A positive correlation between the frequency of Helios+ Treg cells in the peripheral blood and disease activity has been observed in patients with systemic lupus erythematosus (SLE) [40,41]. In this study, we analyzed the expression of Ikaros transcription factors in Treg cells, Th1 cells and Th17 cells from patients with RA and we explored possible associations between their expression levels and disease activity.

## 2. Methods

### 2.1. Blood Samples

Peripheral blood was drawn from RA patients and healthy controls in the outpatient clinic of the University Hospital Cologne. All patients fulfilled the 2010 ACR/EULAR classification criteria. Written informed consent was obtained before blood was drawn in accordance with the Declaration of Helsinki. The study was approved by the Ethics Committee of the University Hospital Cologne (no. 13-091). 

### 2.2. Isolation of Primary CD4^+^ T-Cells 

Peripheral blood mononuclear cells (PBMC) were isolated using density gradient centrifugation (Pan Biotech, Aidenbach, Germany). CD4+ T-cells were isolated using an MACS T-cell isolation kit (Miltenyi Biotech, Bergisch Gladbach, Germany). Cell numbers were assessed using the CellCountess (Life Technologies GmbH, Darmstadt, Germany). CD4+ T cells were purified by negative selection using the CD4+ T cell isolation kit using the QuadroMACS device (all Miltenyi Biotec, Bergisch Gladbach, Germany) and purity of cell suspension was at least 96% and was verified by flow cytometry. Viable cells were counted using the automated cell counter CellCountess (Life Technologies GmbH, Darmstadt, Germany).

### 2.3. Flow Cytometry

For intracellular staining, CD4+ T cells were permeabilized using the cytofix-permwash kit (BD Biosciences, Heidelberg, Germany). Cell viability was assessed by Life-Dead Staining (ThermoFisher Scientific, Schwerte, Germany). Antibodies against Ikaros, Helios, Aiolos, Eos, CD4+, CD25, CD127, FoxP3, IFN-γ, anti-IL-17 antibodies were purchased from BD Biosciences (Heidelberg, Germany). Flow cytometry analysis was performed using the Gallios 10/3 flow cytometer (Beckman Coulter, Krefeld, Germany). The mean fluorescence intensity (MFI) ratio was calculated by dividing the MFI of an antigen by the MFI of its isotype control.

### 2.4. Statistical Analysis

Statistical analysis was performed using SPSS. Where indicated, data were analyzed by non-parametric a Mann–Whitney U test or one-way ANOVA with Tukey’s multiple comparison test and are presented as the median with interquartile ranges (IQR) or mean +/− SEM. Correlation was calculated using Spearman’s Rho test. *p* < 0.05 was considered as statistically significant.

## 3. Results

### 3.1. Ikaros Zinc Finger Transcription Factors in Treg Cells from RA Patients

**Figure 1:**
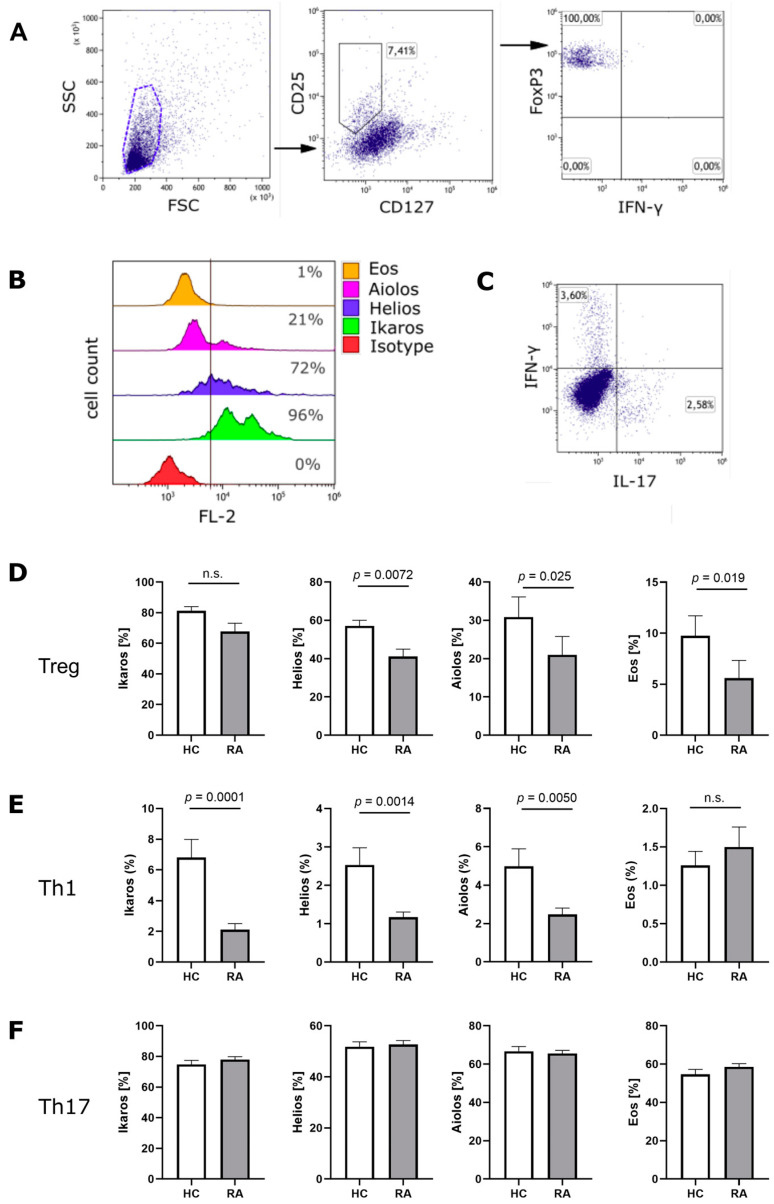
Ikaros zinc finger transcription factor expression in Treg cells, Th1 cells and Th17 cells from patients with RA. (**A**) Gating strategy for the identification CD127^low^CD25^high^FoxP3^+^ Treg cells. (**B**) Representative example of flow cytometry analysis of Ikaros, Helios, Aiolos and Eos in Treg cells from RA patients. (**C**) Representative example of IFN-γ and IL-17 analysis. (**D**) Ex vivo expression of Ikaros zinc finger family members in Treg cells, (**E**) Th1 cells and (**F**) Th17 cells from RA patients (RA; *n* = 20) and healthy controls (HC; *n* = 12). Significance was calculated using non-parametric Mann–Whitney test and results are presented as mean +/− SEM.

We analyzed the expression of Ikaros zinc finger family members on protein level in Treg cells, Th1 cells and Th17 cells from RA patients and compared them with expression levels in healthy individuals. The patients’ characteristics are summarized in Table 1. PBMCs were isolated from the peripheral blood and CD4+ T cells were purified by MACS technique. CD4+ T cells were then analyzed ex vivo by flow cytometry. Figure 1A shows the gating strategy used to identify CD25^high^CD127^low^FoxP3^+^ Treg cells. A representative example of Ikaros, Helios, Aiolos and Eos staining in Treg cells is shown in Figure 1B and a representative example of IFN-γ and IL-17 staining is presented in Figure 1C. We found a substantial decrease in Helios (41.08% vs. 57.08%, *p =* 0.0072), Aiolos (20.97% vs. 30.84%, *p =* 0.025) and Eos (5.60% vs. 9.75%, *p =* 0.019) expression in Treg cells from RA patients as compared to healthy controls (Figure 1D). In Th1 cells from RA patients, the expression level of Ikaros (2.11% vs. 6.81%, *p =* 0.0001), Helios (1.17% vs. 2.53%, *p =* 0.0014) and Aiolos (2.48% vs. 4.98%, *p =* 0.0050) were significantly reduced (Figure 1E). In contrast, there was no difference in Ikaros, Helios, Aiolos or Eos expression between Th17 cells from RA patients and Th17 cells from healthy individuals (Figure 1F).

### 3.2. Correlation between Disease Activity and Aiolos or Helios Expression in Treg Cells

**Figure 2:**
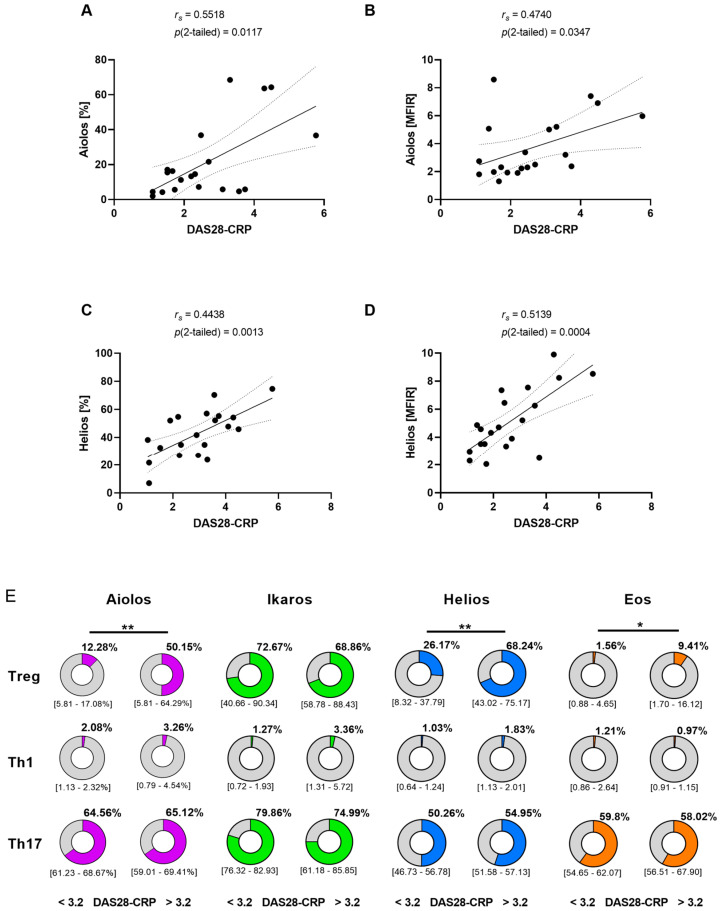
Correlation between disease activity and Aiolos or Helios expression in Treg cells. (**A**) Correlation between Aiolos expression (%) in Treg cells and DAS28-CRP in patients with RA (*n* = 20). (**B**) Correlation between mean fluorescence intensity ratio (MFIR) of Aiolos in Treg cells with DAS28-CRP in patients with RA (*n* = 20). (**C**) Correlation between Helios expression (%) in Treg cells and DAS28-CRP in patients with RA (*n* = 20). (**D**) Correlation between mean fluorescence intensity ratio (MFIR) of Helios in Treg cells with DAS28-CRP in patients with RA (*n* = 20). (**E**) Percentage of Aiolos, Ikaros, Helios or Eos expressing Treg cells, Th1 cells and Th17 cells in the peripheral blood of patients with low disease activity (DAS28-CRP ≤ 3.2) or high disease activity (DAS28-CRP > 3.2) (*n* = 20). Significance was calculated by Spearman’s Rho test or by one-way ANOVA with Tukey’s multiple comparison test. Where indicated, results are presented as the median with interquartile ranges (IQR) (* *p* < 0.05, ** *p* < 0.01).

To explore how disease activity affects the expression of Ikaros zinc finger proteins in RA, we compared our flow cytometry results with the DAS28-CRP scores of the patients. Interestingly, we observed a correlation between Aiolos expression in Treg cells and disease activity (*r_s_* = 0.5518, *p*(2-tailed = 0.0117)) (Figure 2A). In addition, the mean fluorescence intensity ratio (MFIR) of Aiolos was associated with disease activity (*r_s_* = 0.4740, *p*(2-tailed = 0.0347)) (Figure 2B). Similarly, the frequency of Helios positive Treg cells (*r_s_* = 0.4438, *p*(2-tailed = 0.0013)) and the MFIR of Helios (*r_s_* = 0.5139, *p*(2-tailed = 0.0004)) was correlated with disease activity (Figure 2C,D). Interestingly, only Aiolos and Helios expression in Treg cells were clearly correlated with disease activity. In addition, we found a weak association between Eos expression in Treg cells and DAS28-CRP (Figure 2E). In contrast to Treg cells, Ikaros transcription factor expression in Th1 and Th17 cells were not associated with disease activity.

## 4. Discussion

The transcription factor Aiolos was first described by Morgan et al. in 1997 as an Ikaros homologue that heterodimerizes with Ikaros proteins [42]. Further research identified Aiolos as an important promotor of Th17 cell differentiation and Treg cell induction [10,13,14]. In our study, we observed an association between Aiolos expression levels in Treg cells and disease activity. Treg cells require IL-2 for induction and maintenance of their suppressive function [43]. Importantly, Aiolos promotes Th17 cell differentiation by silencing *IL2* [10]. Therefore, we speculate that reduced expression of Aiolos might prevent transdifferentiation of Treg cells into Th17 cells, thereby leading to reduced disease activity in RA. The upregulation of Aiolos in Treg cells from patients with high disease activity might reflect the shift of the Th17/Treg cell ratio towards Th17 cells that is observed in patients with autoimmune diseases and high disease activity [44]. In this context, Helios might be upregulated as a counter-regulatory mechanism.

Ikaros is a negative regulator of Th1 cell differentiation. Therefore, the observed downregulation of Ikaros in Th1 cells represents a potential mechanism of enhanced Th1 cell activity and elevated IFN-γ levels in RA. Th1 cells play a crucial role in RA pathogenesis, and their differentiation is inhibited by Ikaros, which silences *T-bet* [16,45]. The Th1 cell phenotype might be further stabilized by reduced levels of Aiolos and Helios in Th1 cells from RA patients. On the other hand, reduced expression of Helios, Aiolos and Eos in Treg cells from RA patients might contribute to reduced Treg cell numbers and diminished Treg cell activity in RA as these Ikaros transcription factors play an important role in the stabilization of the Treg cell phenotype.

Our findings reveal that Aiolos and Helios expression correlate with disease activity and might therefore be potential biomarkers for disease activity in RA. Unspecific inflammatory markers such as CRP, ESR and IL-6 are elevated during infections, in malignant diseases and other systemic inflammatory conditions. Moreover, clinical assessment of disease activity can be hampered by activated osteoarthritis or other medical conditions, such as fibromyalgia. Therefore, a specific laboratory parameter for disease activity would be very helpful in daily clinical practice. So far, different panels of inflammatory proteins have been studied for their ability to measure disease activity in RA [46]. However, no RA specific laboratory parameter has been established as a surrogate marker for disease activity in daily clinical practice. The Ikaros transcription factors Helios and Aiolos could therefore be helpful for assessing disease activity with laboratory tests.

## Figures and Tables

**Table 1:** Patients’ characteristics.

	RA (*n* = 20)	HC (*n* = 12)
Age	57.5 ± 6.27	52.9 ± 4.14
Sex (%female)	68.2%	67.5%
Disease duration ^1^	6.3 ± 4.9	n/a
Treatment ^2^		
cDMARDs	6	n/a
bDMARDs	8	
tsDMARDs	5	
untreated	1	
DAS28-CRP	3.1 ± 2.2	n/a
RF+	70%	n/a
ACPA+	67.5%	n/a
CRP	11.8 ± 4.2	n/a
ESR	19.8 ± 6.9	n/a

RA, rheumatoid arthritis; HC, healthy control; DAS28-CRP, disease activity score of 28 joints based on CRP; RF, rheumatoid factor; ACPA, anti-citrullinated protein; CRP, c-reactive protein (mg/L); ESR, erythrocyte sedimentation rate (mm/h); n.a., not applicable; mean values ± SEM are shown; ^1^ years; ^2^ number of patients currently treated with DMARDs.

## Data Availability

Not applicable.

## Sample Availability

All data used to support the findings of this study are available from the corresponding author upon reasonable request.

## Abbreviations

Treg cells, regulatory T cells; RA, rheumatoid arthritis; IKZF, Ikaros zinc finger family; PBMC, peripheral blood mononuclear cells; FoxP3, factor forkhead box P3; CRP, c-reactive protein; ESR, erythrocyte sedimentation rate; DAS28, disease activity score 28.
